# Lactic Acid Bacteria Mixture Isolated From Wild Pig Alleviated the Gut Inflammation of Mice Challenged by *Escherichia coli*

**DOI:** 10.3389/fimmu.2022.822754

**Published:** 2022-01-26

**Authors:** Yifan Zhong, Dongyan Fu, Zhaoxi Deng, Wenjie Tang, Jiangdi Mao, Tao Zhu, Yu Zhang, Jianxin Liu, Haifeng Wang

**Affiliations:** College of Animal Science, Zhejiang University, The Key Laboratory of Molecular Animal Nutrition, Ministry of Education, Hangzhou, China

**Keywords:** wild pig, lactic acid bacteria, gut microbiota, immunomodulation, gut health

## Abstract

Wild pigs usually showed high tolerance and resistance to several diseases in the wild environment, suggesting that the gut bacteria of wild pigs could be a good source for discovering potential probiotic strains. In our study, wild pig feces were sequenced and showed a higher relative abundance of the genus *Lactobacillus* (43.61% vs. 2.01%) than that in the domestic pig. A total of 11 lactic acid bacteria (LAB) strains including two *L. rhamnosus*, six *L. mucosae*, one *L. fermentum*, one *L. delbrueckii*, and one *Enterococcus faecalis* species were isolated. To investigate the synergistic effects of mixed probiotics strains, the mixture of 11 LAB strains from an intestinal ecology system was orally administrated in mice for 3 weeks, then the mice were challenged with Escherichia coli ATCC 25922 (2 × 10^9^ CFU) and euthanized after challenge. Mice administrated with LAB strains showed higher (*p* < 0.05) LAB counts in feces and ileum. Moreover, alterations of specific bacterial genera occurred, including the higher (*p* < 0.05) relative abundance of *Butyricicoccus* and *Clostridium* IV and the lower (*p* < 0.05) abundance of *Enterorhabdus* in mice fed with mixed LAB strains. Mice challenged with *Escherichia coli* showed vacuolization of the liver, lower GSH in serum, and lower villus to the crypt proportion and Claudin-3 level in the gut. In contrast, administration of mixed LAB strains attenuated inflammation of the liver and gut, especially the lowered IL-6 and IL-1β levels (*p* < 0.05) in the gut. Our study highlighted the importance of gut bacterial diversity and the immunomodulation effects of LAB strains mixture from wild pig in gut health.

## 1 Introduction

Homeostasis of gut health, together with the diverse and complex microbial community harbored in the gut, plays a central role in host health (1). Disorder of gut health, including the alteration of gut microbiota, impairment of barrier function, and disruption of the immune system, further lead to several diseases of the host (2). Supplementation of probiotics has been revealed as one of the effective strategies to maintain the gut health (3). According to the definition, probiotics are “living microorganisms that confer several health benefits when administrated in adequate amounts to the host” (4). Most often used as probiotic supplements, lactic acid bacteria (LAB) include many bacterial genera, including *Lactobacilli*, *Lactococci*, *Enterococci*, *Streptococci*, *Leuconostoc*, and *Pediococci*, among which the best known is the genus *Lactobacillus* (5). Numerous studies have revealed the beneficial effect in applying LAB, with the mechanisms behind including suppression of pathogens, manipulation of microbiota communities, immunomodulation, stimulation of epithelial cell proliferation, and differentiation and fortification of the gut barrier (6).

Isolation and characterization of bacterial strains was the first step in the discovery and application of probiotics. As the gut is one of the sources for probiotic strains, probiotics such as *L. gasseri*, *L. reuteri*, and *L. fermentum* isolated from the human gut exert therapeutic and protective activities (7). However, imbalance in the gut microbiota usually occurs in humans with industrialized lifestyles, following a rise in diseases such as allergic and autoimmune disorders, and inflammatory bowel disease, indicating the importance of preserving the treasure of microbial diversity from rural communities (8). On the other hand, the gut of pigs is also suggested as a good source of probiotics (9). In our previous study, probiotic strain *L. reuteri* ZJ617 was isolated from the domestic pig intestine and showed high adhesive ability together with inhibition activity against pathogens including *Escherichia coli* K88 and *Salmonella enteritidis* 50335 (10). Compared with the domestic pig fed with a commercial diet, wild pigs live in a wild environment mainly fed on a diet that includes acorns, wild fruits, grassroots, and stems with high cellulose content and low carbohydrate or fat content (11). It has been previously reported that a strong distinction existed in the bacterial diversity of wild pigs and domestic pigs. What is more, predictions of metagenome function showed more bacterial genes related to the immune system and environmental adaptation in the feces of wild pigs than that in domestic pigs (12). To investigate the probiotics in wild pigs, Li and colleagues assessed the probiotic characteristics and safety properties of LAB strains isolated from the gut, including *L. mucosae*, *L. salivarius*, *Enterococcus hirae*, *Enterococcus durans*, and *Enterococcus faecium* (13). Previous studies have suggested the beneficial effects of probiotics in the gut of wild pigs, while few studies demonstrated the mechanism behind applying probiotics strains in gut health. On the other hand, although beneficial effects of single-strain probiotics to health were revealed in numerous studies, the potential of synergistic effects from mixed probiotics strains is still not fully explored (14).

In this study, we sequenced and isolated LAB strains from the feces of a wild pig, following administrated LAB strain mixture in mice challenged with *E. coli*, aiming to investigate the synergistic effects of probiotic strains and illustrate the mechanisms in the contribution to gut health.

## 2 Materials and Methods

### 2.1 Animals and Fecal Collection

In this study, one adult WP weighting 100 kg from a wild environment and three adult DP (Duroc × Landrace × Yorkshire) with similar weights were selected. Fecal pellets were collected and stored at -80°C immediately with 20% glycerol for further analysis.

### 2.2 16S rRNA Gene Amplicon Sequencing

Amplicon sequencing of the 16S RNA was performed by Realbio Genomics Institute (Shanghai, China). Briefly, DNA was extracted and the V3–V4 region was amplified in PCR reactions using primers 341F: 5′-CCTACGGGRSGCAGCAG-3′ and 806R: 5′-GGACTACVVGGGTATCTAATC-3′ and sequenced on a HiSeq platform (Illumina Inc., CA, USA) for paired-end reads of 250 bp. Reads were clustered into Operational Taxonomic Units (OTUs) with 97% similarity (15) and classified with RDP Classifier (http://rdp.cme.msu.edu/). QIIME1 (v1.9.1) was used in the OTU profiling and alpha/beta diversity analyses. All DNA sequences in this study were deposited in the NCBI sequence read archive with the project number PRJNA778598.

### 2.3 Isolation and Characterization of LAB Strains

#### 2.3.1 Isolation of LAB Strains

LAB strains were isolated according to methods described in a previous study (10). Briefly, feces from WP was suspended, homogenized, and spread on de Man, Rogosa and Sharpe (MRS) agar (Qingdao Haibo Bio, China) plates and incubated anaerobically. After incubation, the white colony of LAB was streaked onto MRS agar plates again and incubated at 37°C for 48 h. Samples from LAB medium plates were transferred to a tube containing 10 ml MRS broth and preserved at -80°C with a dilution of 40% (w/v) sterile glycerol for further use.

#### 2.3.2 Sequencing and Phylogenetic Analysis of LAB

DNA of the LAB strains was extracted, and full lengths of 16S rRNA genes were amplified with the following primers: forward 5′-A GAGTTTGATCCTGGCTCAG-3′ and reverse 5′-GGTTACCTTGTTACGACTT-3′ (16). The PCR products were sequenced (Shangya Biotechnology, Hangzhou, China), and sequences were compared with the sequences available in the NCBI of BLAST program (https://blast.ncbi.nlm.nih.gov/Blast.cgi). Phylogenetic tree analysis was achieved with MEGA 7.0 (http://megasoftware.net/). Sequences were submitted in GenBank, and the name and accession numbers (MT12247, MT712248, MT712249-MT712254, MT712255, MT712256, MT712257) are obtained.

#### 2.3.3 Characterization of LAB Strains

LAB strains were characterized according to the procedures in a previous study (10). Briefly, bacterial culture inoculated into MRS broth was measured for the growth curve in 0, 6, 9, 12, 15, 18, 21, and 24 h. Auto-aggregation assay (17) was applied, and absorbance of the supernatant was measured to determine the specific cell–cell interactions. In terms of cell surface hydrophobicity, xylene was added to the cell suspension and the aqueous phase was measured. All the absorbance of bacterial culture, supernatant, and aqueous phase was measured for each time at 600 nm using a BioTek Synergy HTX multi-mode reader (Thermo Fisher Scientific, Waltham, MA, USA). Acid and bile salt survivability of the LAB was assessed in the MRS broths with pH = 3.0 or 0.1% bile salt. Viable bacterial counts were determined by plating appropriate dilutions on MRS agar medium. For the tolerance of Cu^2+^ and Zn^2+^, MRS mediums were prepared with 100 mg/L or without Cu^2+^/Zn^2+^ and inoculated with 2% of culture and incubated for 24 h at 37°C. Optical density at OD 600 nm was measured for monitoring the growth kinetics. The above tests were carried out in triplicate for each strain.

### 2.4 Oral Administration of LAB and Sample Collection in Mouse Study

All animal experiments were performed in accordance with the guidelines for the care and use of laboratory animals approved by the Institutional Animal Care and Use Committee of Zhejiang University (No. 20170529). In this study, a total of 36 male C57BL/6J mice were purchased from Shanghai SLAC Laboratory Animal Co., Ltd. (Shanghai, China) and fed with chow diet *ad libitum*. Mice were housed in a quiet and ventilated environment at 25°C, 50% humidity, and 12-h light–dark cycle.

As shown in **Figure S1A**, mice were randomly divided into four groups of nine mice each. Four groups were treated as follows: (1) mice orally administrated with 200 μl of PBS for 3 weeks (C); (2) mice orally administrated with 200 μl of PBS for 3 weeks followed by oral challenge with *E. coli* ATCC 25922 (18) as gut inflammatory model (2 × 10^9^ CFU) (CE); (3) mice orally administrated with mixed LAB (2 × 10^9^ CFU) for 3 weeks (M); and (4) mice orally administrated with mixed LAB (2 × 10^9^ CFU) for 3 weeks followed by oral challenge with *E. coli* ATCC 25922 (2 × 10^9^ CFU) (ME), respectively. The body weight of all mice was recorded daily, and feces of all mice was collected aseptically and suspended into sterile saline to determine viable LAB counts. At the endpoint, the body temperature of each mouse was measured, and blood and fecal samples were collected before euthanasia. Viable LAB that adhered in the jejunum and ileum epithelium were counted by plating appropriate dilutions on MRS agar medium.

### 2.5 Biochemical Assays of Serum

The concentrations of T-AOC, SOD, GSH, DAO, TNF-α, IL-1β, and IL-6 in the serum were determined with commercially available ELISA kits according to the protocol provided by the manufacturer (Nanjing Jiancheng Bioengineering Institution).

### 2.6 Hematoxylin and Eosin Staining of Liver and Gut

Hematoxylin and eosin (H&E) staining of liver and gut tissues was performed as previously described (19). In brief, samples from the liver, ileum, and colon were soaked in 4% paraformaldehyde, waxed, and sliced into 5 µm-thick sections. After deparaffinization and dehydration, sections were soaked in graded alcohols and stained with H&E subsequently. Photomicrographs were obtained *via* optical microscopy, and crypt length was measured using Imaging Software (CS-EN-V1.18) (Olympus Corporation).

### 2.7 Western Blotting

Bradford’s method was applied in the determination of protein concentration in samples (20). Protein samples were loaded on sodium dodecyl sulfate–polyacrylamide gel electrophoresis (SDS-PAGE) and transferred to polyvinylidene fluoride (PVDF) membranes. The primary antibody was applied to incubate and block the membrane at 4°C overnight. The blot was developed with electrochemiluminescence (Millipore) after incubation with the secondary antibody. Bands were measured using ImageJ software (National Institutes of Health) and standardized to the density of GAPDH.

### 2.8 Quantitative Real-Time Polymerase Chain Reaction Analysis

Quantitative real-time polymerase chain reaction analysis (qRT-PCR) of mRNA from the gut was performed with TB Green Premix Ex Taq (Tiangen Biotech) according to the instructions. The sequences for PCR primers were as follows: GAPDH (5′-CGCGAGAAGATGACCCAGAT-3′, 5′-GCACTGTGTTGGCGTACAGG-3′); TNF-α (5′-CGTTGTAGCCAATGTCAAAGCC-3′, 5′-TGCCCAGATTCAGCAAAGTCCA-3′); IL-1β (5′-TCTTTGAAGTTGACGGACCC-3′, 5′-TGAGTGATACTGCCTGCCTG-3′); IL-6 (5′-GCTACCAAACTGGATATAATCAGGA-3′, 5′-CCAGGTAGCTATGGTACTCCAGAA-3′). The data obtained were analyzed using the Mx3000P system (Agilent), and β-actin was used as internal standard in all gene quantifications performed. The final data were derived from the formula 2^-ΔΔ^Ct.

### 2.9 Gut Microbiota Profiling

The 16S rRNA gene amplicon sequencing of fecal samples in mice was performed. The detailed procedures were described in Section 2.2.

### 2.10 Statistical Analysis

All data were expressed as mean ± standard error of the mean (SEM). Student’s *t*-test, one-way ANOVA, and two-way ANOVA were applied in the comparison of two groups, bacterial strains, and four groups, respectively. For the abundance of gut microbiota, Kruskal–Wallis H test and Dunn’s *post hoc* test were carried out on the comparison among four groups. Analysis of similarities (Anosim) was applied for the beta diversity of gut microbiota between four groups. Linear discriminant analysis effect size (LEfSe) was used to the detect the differential microbiota at the genus level, and the linear discriminant analysis (LDA) score of each microbiota was given. In each case, *p*-values < 0.05 were considered statistically significant. Analysis of the dataset was completed with R software (version 3.5.1).

## 3 Results

### 3.1 Isolation and Identification of LAB Strains From Wild Pig

**Figure 1:**
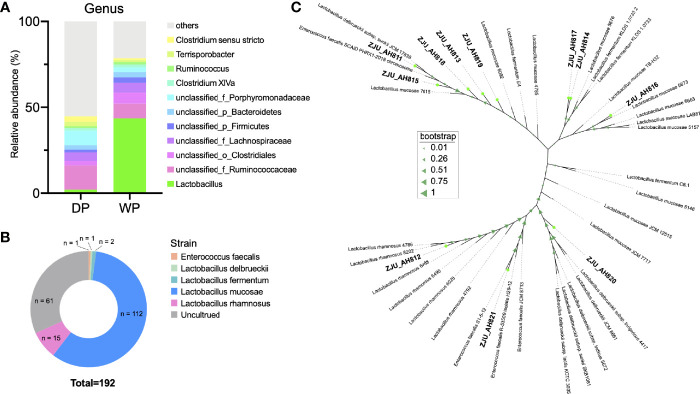
Isolation and identification of lactic acid bacteria (LAB) strains. **(A)** Relative abundance (>1%) of gut microbiota at the genus level between domestic pig (DP) and wild pig (WP). **(B)** Proportion of the cultured LAB strains isolated from WP. **(C)** Phylogenetic tree of 11 LAB strains with similarity < 97% (ZJU_AH811, ZJU_AH812, ZJU_AH813, ZJU_AH814, ZJU_AH815, ZJU_AH816, ZJU_AH817, ZJU_AH818, ZJU_AH819, ZJU_AH820, ZJU_AH821) and reference sequences from NCBI.

Results of 16S rRNA gene sequencing (**Figure 1A** and **Table S1**) revealed high relative abundance of *Lactobacillus* in feces of WP (43.61%) than that in DP (2.01%). When LAB strains from WP were isolated and cultured (**Figure 1B**), a total of 112, 15, 2, 1, and 1 strains were classified as *L. mucosae*, *L. rhamnosus*, *L. fermentum*, *L. delbrueckii*, and *Enterococcus faecalis* within the 192 cultures, respectively. According to the threshold of 97% similarity, 11 LAB strains were identified, namely, ZJU_AH811, ZJU_AH812, ZJU_AH813, ZJU_AH814, ZJU_AH815, ZJU_AH816, ZJU_ AH817, ZJU_AH818, ZJU_AH819, ZJU_AH820, and ZJU_AH821. As shown in **Figure 1C**, a phylogenetic tree of 11 LAB strains with reference sequences from NCBI was constructed. Within the 11 LAB strains, ZJU_AH811 and ZJU_AH812 belong to *L. rhamnosus*, ZJU_AH819 belongs to *L. fermentum*, ZJU_AH820 belongs to *L. delbrueckii*, ZJU_AH821 belongs to *Enterococcus faecalis*, and the rest of the 6 strains belong to *L. mucosae.*

### 3.2 Characteristics of LAB Strains

**Figure 2:**
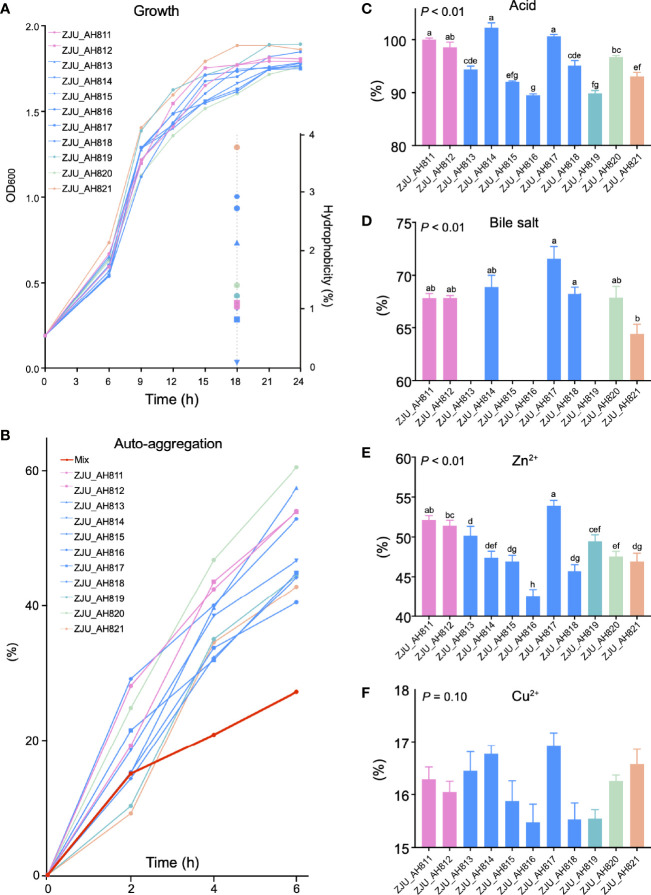
Characteristics of LAB strains. Growth curve and hydrophobicity **(A)**, and auto-aggregation **(B)** of single and mixed of 11 LAB strains. Tolerance of **(C)** acid, **(D)** bile acid, **(E)** Zn^2+^, and **(F)** Cu^2+^ of 11 LAB strains (ZJU_AH811, ZJU_AH812, ZJU_AH813, ZJU_AH814, ZJU_AH815, ZJU_AH816, ZJU_AH817, ZJU_AH818, ZJU_AH819, ZJU_AH820, ZJU_AH821). Results are expressed as mean ± standard error of the mean (SEM). Letters a-h in the same graphic were significantly different (p < 0.05).

As the LAB strains were isolated and identified from WP, the growth kinetics of the 11 LAB strains were evaluated (**Figure 2A**). After 24 h, ZJU_AH819 and ZJU_AH817 showed the highest growth rate value indicated as OD_600 nm_ values, respectively. The hydrophobicity of LAB strains was tested at 18 h (**Figure 2A**), and ZJU_AH821 and ZJU_AH814 showed the highest (3.76%) and lowest (0.08%) hydrophobicity among the 11 LAB strains. The auto-aggregation curves within 6 h of 11 LAB strains alone or mixed were generated (**Figure 2B**), which ranged from 60.54% to 27.24%. The mixed LAB strains showed the lowest auto-aggregation than 11 LAB strains alone. All the 11 LAB strains tested showed tolerance to acid (pH = 3), and the survival rate of LAB strains was between 102.25 ± 0.95% and 89.48 ± 0.32% after 3 h of exposure to acid (**Figure 2C**, *p* < 0.01). The survivability in 0.3% bile salt was examined (**Figure 2D**, *p* < 0.01), and seven LAB strains were able to survive with a survival rate ranging from 64.43 ± 0.92% to 71.58 ± 1.13% after a 3-h exposure, including ZJU_AH811, ZJU_AH812, ZJU_AH814, ZJU_AH817, ZJU_AH818, ZJU_AH820, and ZJU_AH821. The results of the LAB strains’ tolerance to 100 mg/l Zn^2+^ or Cu^2+^ are shown in **Figures 2E, F**. The LAB strains ZJU_AH817 and ZJU_AH816 showed the highest (53.93%) and the lowest (42.57%) tolerance to Zn^2+^ after 24 h, respectively. The tolerance of Cu^2+^ showed no significant difference between 11 LAB strains (*p* = 0.01).

### 3.3 Adherence of Mixed LAB Strains in the Gut and Composition of Gut Microbiota in Mice

**Figure 3:**
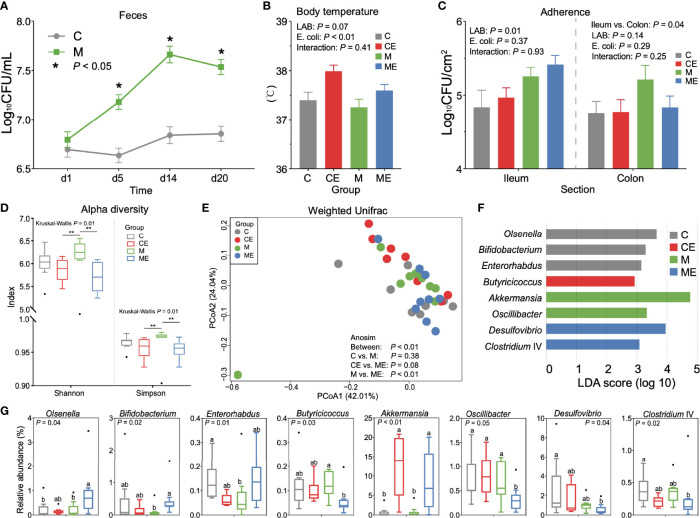
Gut microbiota profiles in mice fed with mixed LAB strains and challenged with *Escherichia coli*. **(A)** Counts of LAB in feces of mice fed with mixed LAB strains (M) and control group (C) at days 1, 5, 14, and 20. **(B)** Body temperature of control mice (C), control mice challenged with *Escherichia coli* (CE), mice fed mixed LAB strains (M), and mice fed mixed LAB strains and challenged with *Escherichia coli* (ME) at the end point. **(C)** Adherence of LAB in the ileum and colon of mice between C, CE, M, and ME groups. **(D)** Alpha diversity (Shannon and Simpson index) of gut microbiota in mice between C, CE, M, and ME groups. **(E)** Principal coordinate analysis (PCoA) on the weighted UniFrac distance matrix of gut microbiota between C, CE, M, and ME groups. **(F)** Linear discriminant analysis (LDA) score of gut microbiota (with LDA score > 2) at the genus level between C, CE, M, and ME groups. **(G)** Relative abundance of bacteria genera with LDA score > 2 between C, CE, M, and ME groups. *p < 0.05, **p < 0.01. Letters a,b in the same graphic were significantly different (*p* < 0.05).

In mice fed with a mixture of 11 LAB strains, feces were collected and cultured to test the viable count of entire LAB in the gut. As shown in **Figure 3A**, the LAB counts in mice fed with mixed LAB strains (M) were 7.18 ± 0.30, 7.66 ± 0.35, and 7.54 ± 0.32 Log_10_ CFU/ml and were significantly higher (*p* < 0.05) than 6.63 ± 0.29, 6.84 ± 0.35, and 6.85 ± 0.31 Log_10_ CFU/ml in the control group (C) at days 5, 14, and 20, respectively. During the study, no significant of feed intake, weight gain, or feed-to-gain ratio was observed between C and M mice (**Figure S1B**). As shown in **Figure 3B**, the challenge with *E. coli* showed a significant effect (*p* < 0.01) on body temperature, while the administration of mixed LAB strains (*p* = 0.07) showed a decreasing trend in the body temperature, and the interaction between mixed LAB strains and *E. coli* showed no significant effect (*p* = 0.41) on the body temperature in mice. As shown in **Figure 3C**, administration of mixed LAB strains showed a significant effect on the counting number of LAB in the ileum (*p* < 0.01) than that in control mice. The challenge of *E. coli* and the interaction between mixed LAB strains showed no effect on the counting number of LAB both in the ileum and colon.

When fecal samples were sequenced, significant differences (*p* = 0.01) of the Shannon and Simpson indices were observed between four groups (**Figure 3D**). Mice fed mixed LAB strains showed both higher Shannon and Simpson indices in the gut microbiota than did CE and ME mice. The PCoA analysis based on the weight UniFrac distance matrix of the gut microbiota revealed the beta diversity in C, CE, M, and ME mice (**Figure 3E**). Analysis of similarities (Anosim) showed no significant difference between the C and M (*p* = 0.38) or CE and ME (*p* = 0.08) group while the profiles of gut microbiota in M and ME mice showed a significant difference (*p* < 0.01). The relative abundance of gut microbiota between C, CE, M, and ME groups at the phylum and genus levels is shown in **Tables S2**, **S3**. LEfSe analysis revealed 8 differential genera (LDA score > 2, **Figure 3F**), and the relative abundance of differential genera between four groups is shown in **Figure 3G**, including *Olsenella*, *Bifidobacterium*, *Enterorhabdus*, *Butyricicoccus*, *Akkermansia*, *Oscillibacter*, *Desulfovibrio*, and *Clostridium* IV. When mice were challenged with *E. coli*, higher (*p* < 0.01) abundance of *Akkermansia* was observed in the CE and ME group than in the C and M group. Mice fed with mixed LAB strains challenged the lower (*p* = 0.05) relative abundance of *Oscillibacter* in the gut than did other groups.

### 3.4 Alleviation of Liver and Gut Morphology in Mice Fed Mixed LAB Strains and Challenged With *E. coli*

**Figure 4:**
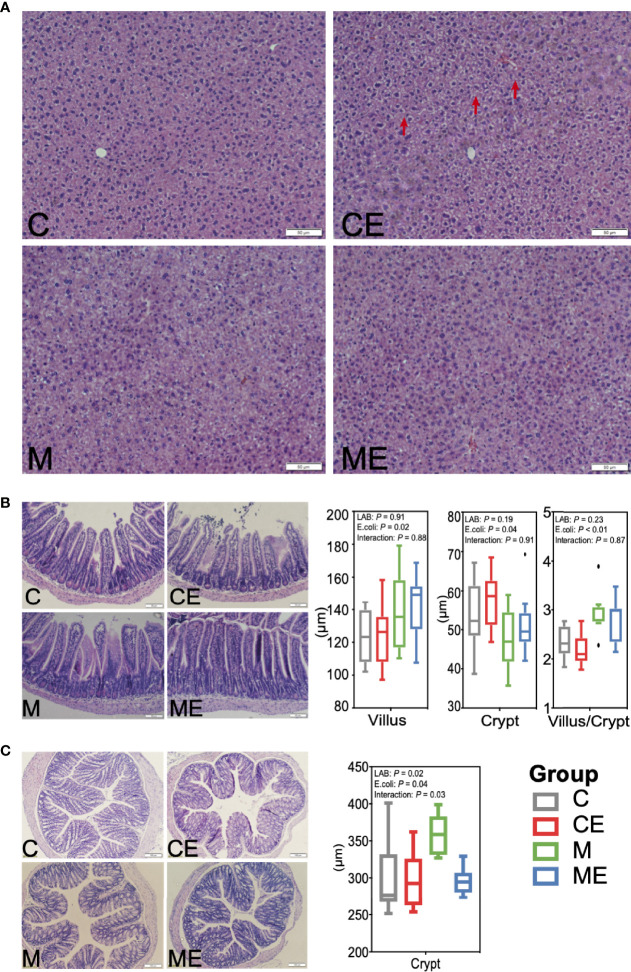
Morphology of the **(A)** liver, **(B)** ileum, and **(C)** colon in control mice (C), control mice challenged with *Escherichia coli* (CE), mice fed mixed LAB strains (M), and mice fed mixed LAB strains and challenged with *Escherichia coli* (ME) at the end point.

H&E staining revealed hepatic vacuolization in CE mice challenged with *E. coli*, while administration of mixed LAB strains attenuated the vacuolization in ME mice (**Figure 4A**). Morphology of the ileum and colon in C, CE, M, and ME mice was also assessed and is shown in **Figures 4B, C**. Administration of mixed LAB strains showed no effect on the villus length (*p* = 0.91) and crypt depth (*p* = 0.19) while challenge with *E. coli* significantly increased the villus length (*p* = 0.02) and crypt depth (*p* = 0.04), and no interaction between LAB and *E. coli* was observed (**Figures 4B, C** **)**. In addition, a lower (*p* < 0.01) proportion of villus to crypt in mice challenged with *E. coli* was observed (**Figure 4B**). In the colon, both LAB (*p* = 0.02) and *E. coli* (*p* = 0.04) showed a significant effect on the crypt depth. Mice administrated with mixed LAB strains showed the highest crypt depth (358.21 µm) than other C (299.73 µm), CE (298.10 µm), and ME (295.74 µm) mice. The interaction between LAB and *E. coli* (*p* = 0.03) was also observed (**Figure 4C**).

### 3.5 Effects of Mixed LAB Strains in the Inflammation of Serum and Gut in Mice

**Figure 5:**
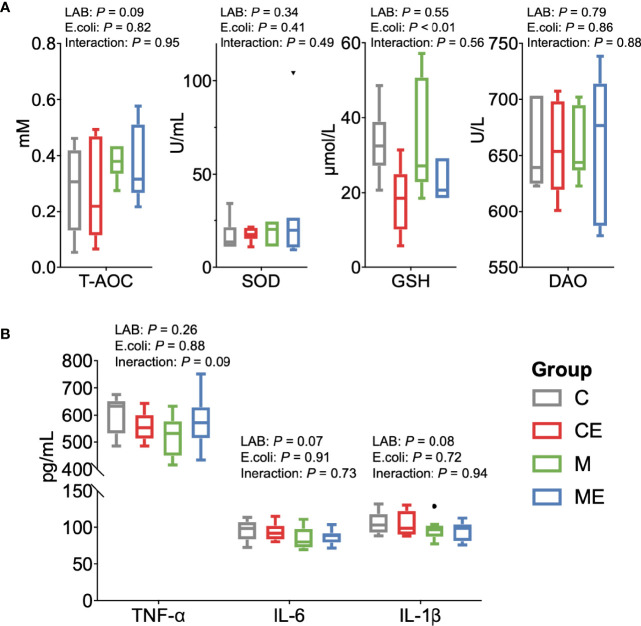
Oxidant and inflammation status of the serum in control mice (C), control mice challenged with *Escherichia coli* (CE), mice fed mixed LAB strains (M), and mice fed mixed LAB strains and challenged with *Escherichia coli* (ME). **(A)** Oxidative stress parameters and **(B)** cytokines in the serum of mice between four groups. T-AOC, total antioxidant capacity colorimetric; SOD, superoxidase dismutase; GSH, glutathione; DAO, diamine oxidase; TNF-α, tumor necrosis factor alpha; IL, interleukin.

**Figure 6:**
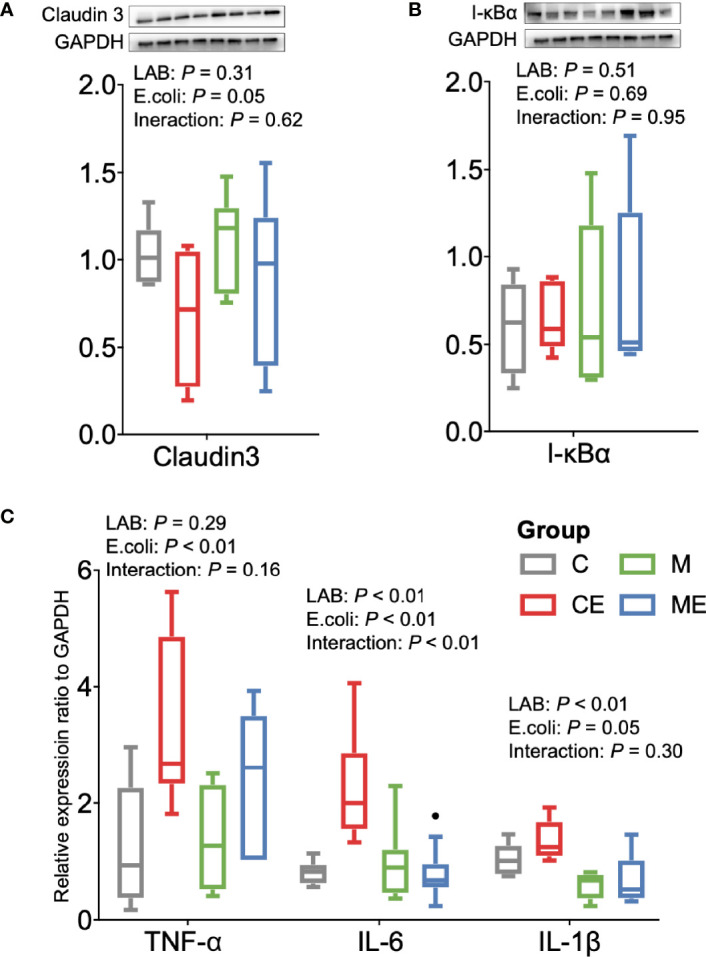
Inflammation status of the gut in control mice (C), control mice challenged with *Escherichia coli* (CE), mice fed mixed LAB strains (M), and mice fed mixed LAB strains and challenged with *Escherichia coli* (ME). Ratio of **(A)** Claudin3, **(B)** I-κBα, and **(C)** mRNA expression of cytokines in the gut of mice between four groups. GAPDH, glyceraldehyde-3-phosphate dehydrogenase; I-κBα, nuclear factor of kappa light polypeptide gene enhancer in B-cells inhibitor, alpha; TNF-α, tumor necrosis factor alpha; IL, interleukin.

The oxidative stress of mice was assessed (**Figure 5A**); the LAB, *E. coli*, or both showed no significant (*p* > 0.05) effect on the T-AOC and SOD levels in serum between C, CE, M, and ME mice. CE and ME mice challenged with *E. coli* showed a significantly lower (*p* < 0.01) GSH level in the serum than that in C and M mice. When cytokines in the serum were measured (**Figure 5B**), LAB or *E. coli* showed no significant (*p* > 0.05) effects on the concentrations of TNF-α, IL-6, and IL-1β in the serum between four groups. In the gut, administration of mixed LAB strains or *E. coli* also showed no significant (*p* > 0.05) effects on the proteins claudin-3 and I-κBα between four groups (**Figures 6A, B** **)**. A significant difference of cytokines in the gut was observed between four groups (**Figure 6C**). Challenge with *E. coli* elevated the levels of TNF-α (*p* < 0.01) and IL-1β (*p* < 0.01) in the gut, and administration of LAB significantly lowered the IL-1β (*p* < 0.01) and IL-6 (*p* < 0.01) in the gut of mice. The interaction (*p* < 0.01) of mixed LAB strains and *E. coli* was observed in the IL-6 level of gut between four groups.

## 4 Discussion

In this study, LAB strains from wild pig were isolated and characterized, which showed beneficial effects on the gut health in mice. Our results revealed the probiotics of LAB strains and the mechanisms in mediating immune defenses against *E. coli* in the gut, highlighting the importance of probiotics in healthy individuals.

LAB is known as a probiotic group and is normally found in the gut, which has been widely applied in humans and animals to promote gut health (21, 22). Compared with the domestic pigs, higher abundance of *Lactobacillus* was found in the gut of wild pig in our study, suggesting the potential relationship between LAB and high disease resistance of wild pigs. As a part of LAB, *Lactobacillus* contributes to the growth and reduction of diarrhea and inhibition of pathogens in pigs (23). Thus, wild pigs might benefit from the high abundance of *Lactobacillus* in the gut. However, previous studies usually observed a higher abundance of *Lactobacillus* in the gut of commercial and domestic native pigs than wild pigs (12, 24), which is inconsistent with our results, partly due to the differential environment or geography (25, 26).

When LAB strains were isolated and cultured, a total of 11 LAB strains were identified and assigned as 5 species, including *L. mucosae*, *L. fermentum*, *L. delbrueckii*, *L. rhamnosus*, and *Enterococcus faecalis.* Similar to previous studies, *L. mucosae* and *L. fermentum* were also isolated from wild pigs (13, 27), indicating the widely distributed inhabitants in the pig. As the characteristics of 11 LAB strains were described, ZJU_AH817 showed the lower hydrophobicity but higher tolerance of acid, bile salt, and Zn^2+^ among the 11 strains. Cell-surface hydrophobicity was associated with bacterial attachment to the surface (28, 29), and greater hydrophobicity of bacteria means higher levels of adhesion (30), which was the first step for the LAB strains performing beneficial effects on the host (31). Moreover, the viability and survival of LAB strains were one of the most important parameters in the condition of low pH from the stomach and bile secreted in the intestine (32). Thus, despite the lower hydrophobicity of ZJU_AH817 which might make it difficult to adhere to the gut, higher tolerance of the acid and bile salt could contribute to the existence of ZJU_AH817 in wild pigs for further properties. What is more, differential tolerance of acid, bile salt, and Zn^2+^ between strains (ZJU_AH813, ZJU_AH814, ZJU_AH815, ZJU_AH816, ZJU_AH817, ZJU_AH818) even from the same species *L. mucosae* was observed, suggesting that the variation of bacteria at the strain level also demonstrated differential function in adhesion (33), immunity (34), and diseases (35). The lower auto-aggregation of mixed LAB strains than other strains also suggested the variation between different strains (36). On the other hand, the tolerance of Zn^2+^ and Cu^2+^ indicates the possibility in the application of LAB strains in pigs, since zinc and copper are classified as trace minerals required by pigs and usually supplemented in diet (37).

The efficacy of single-strained and multi-strained mixture probiotics remains debated. McFarland reviewed randomized controlled trials and found a similar efficiency of mixtures and single strains in the prevention or treatment of disease (38), while Chapman and Gibson claimed that the mixtures appear to be more effective against a wide range of end points (39). The synergistic interactions between mixed strains could be the key which influences the efficiency of probiotics (40). Since the LAB strains were isolated together from the wild pig in this study, we believe that the synergistic interactions of 11 LAB strains could exist and be more effective than a single strain. When administrated with the mixed LAB strains, mice showed higher LAB counts in the gut and feces, suggesting the colonization of LAB strains. Probiotic strains could stimulate the enterocyte migration in mice (41, 42); higher crypt depth in mice administrated with LAB strains was observed in our study, which was in accordance with previous studies. Indeed, *Butyricicoccus*, one of the butyrate producers (43), enriched in mice administrated with LAB strains, could also contribute to the crypt depth *via* butyrate in the colon (44). In addition to the colonization and crypt depth, similar feed intake, weight gain, gut microbiota profiles, or serum oxidative parameters between M and C groups were observed, suggesting that colonization of LAB strains showed a limited effect on healthy individuals. Recently, a systemic review also concluded that the probiotic supplementation showed a limited effect on immune and inflammatory markers in healthy adults (45).

Published studies usually focused on metabolic actions of probiotics while paying less attention on the immune response of probiotics. In our study, mixed LAB strains exerted the beneficial effects *via* the immunomodulation effects when inflammation of the gut and liver occurred in mice challenged with *E. coli*. Vacuolization of liver and lower GSH concentrations in the serum was observed, suggesting the oxidative stress triggered by *E. coli* (46). In the gut, higher diversity and differential patterns of microbiota in the M group were observed than those in the CE and ME group. Loss of microbiota diversity appears as a common feature of gut dysbiosis and diseases in several human studies (47, 48), which highlighted the importance of supplementation with probiotics in the improvement of diversity and gut health (49). LEfSe analysis revealed the alteration of specific bacterial genera after challenge with *E. coli* in mice, for instance, higher relative abundance of *Enterorhabdus* and *Akkermansia* was observed in mice challenged with *E. coli*. *Enterorhabdus* isolated from the inflamed ileal mucosa in a mouse (50) were observed to be enriched in the disordered gut of mice fed a Western-style diet or high-fructose intake (51). Lower abundance of *Enterorhabdus* in the gut suggests the improvement of gut health in mice fed with mixed LAB strains. However, *Akkermansia*, one of the next-generation probiotics (52, 53), was enriched in mice challenged with *E. coli*, which was inconsistent with previous studies, and the mechanism behind it requires further investigation. Besides, challenge with *E. coli* lowered the relative abundance of *Clostridium* IV in mice. As one of the major inhabitants in the gut, a decrease in *Clostridium* IV was associated with loss of gut microbiome colonization resistance in patients with chronic gut inflammation compared to healthy subjects (54, 55).

Apart from the gut microbiota, challenge with *E. coli* altered the gut morphology and tight junction, together with increased levels of cytokines, including TNF-α, IL-6, and IL-1β in the gut. The villi, crypts, and villus height-to-crypt depth ratio are critical histomorphometric parameters in the final stage of nutrient digestion and assimilation (56). Human studies showed that *E. coli* could induce damage to epithelial cells (57). Similar to the previous study, a lower trend of a sealing component of tight junction, claudin 3, was also observed in the mice challenged with *E. coli*. What is more, previous studies revealed that damage of epithelial cells induced by *E. coli* also interacted with the elevated levels of pro-inflammatory cytokines in fecal samples from children and adult with diarrhea (58, 59). A study on mice also showed that impairment of the gut might be highly related to the increase in oxidative stress, including the upregulation of the pro-inflammatory cytokines (TNF-α, IL-6, and IL-1β) (60). During the inflammatory response, pro-inflammatory cytokines are produced, which cause disruption of the gut barrier (61). Overexpression of several cytokines in the inflamed gut has been suggested as a contributor to impairment of the gut. For example, *in vitro* studies revealed that TNF-α could decrease the protein expression of the tight-junction proteins (62, 63). In various inflammatory diseases of the gut, IL-6 has been shown to play a critical role and contribute to the pro-inflammatory cascade, which includes barrier disruption (64, 65). Previous studies also showed that IL-1β caused an increase of permeability in the gut both *in vivo* and *in vitro* (66, 67). On the other hand, studies indicated that inhibition of cytokines could exert beneficial effects against intestinal mucosal damage and development of inflammation in the gut (61). However, no significant difference of inflammation cytokines in serum was observed including TNF-α, IL-6, and IL-1β between four groups, indicating that the challenge of *E. coli* ATCC 25922 (18) might induce a local rather than a systemic inflammation in mice.

When challenged with *E. coli* in mice fed with mixed LAB strains, a lower trend of the relative abundance of *Desulfovibrio* was observed. The opportunistic pathogen *Desulfovibrio* enriched in patients with chronic inflammatory processes (68) and oral administration of probiotic strain *L. plantarum* P-8 decreased the abundance of *Desulfovibrio* in adults of different ages (69). Apart from the inhibition of potential pathogens, supplementation of mixed LAB strains also attenuated the inflammation in the liver and gut in mice challenged with *E. coli*. Previous studies also revealed that pretreatment of probiotics *L. reuteri* ZJ617 attenuated the hepatic inflammatory and autophagy through the gut–liver axis both in mice (70) and in piglets (71). After challenge with *E. coli*, lower levels of IL-6 and IL-1β in the gut of mice administrated with mixed LAB strains were observed than those in the control group, indicating the immunomodulation effect of LAB strains isolated from the wild pig. Since the importance of gut microbiota to the immune system has been demonstrated (72), it is urgent to apply probiotics treating various diseases *via* regulation of cytokine profiles in the gut (73).

Taken together, mixed LAB strains from wild pig exerted a beneficial effect on the host *via* immunomodulation of IL-6 and IL-1β against the infection of *E. coli* in the gut while the exact mechanisms behind it, including specific components/metabolites derived from the LAB strains and pathways in the gut, warrant further illustration. Also, other probiotics including bacillus, yeast, and some other bacterium in the gut of wild pigs warrant further investigation in future studies. This study highlighted the importance of preserving bacterial diversity and beneficial effects of LAB strains mixture from wild pig as probiotics in the gut.

## Data Availability Statement

The datasets presented in this study can be found in online repositories. The names of the repository/repositories and accession number(s) can be found in the article/**Supplementary Material**.

## Ethics Statement

The animal study was reviewed and approved by the Institutional Animal Care and Use Committee of Zhejiang University.

## Author Contributions

HW designed the experiments. DF, ZD, WT, JM, TZ, and YuZ performed the experiments. HW, YiZ, DF, and JL analyzed the data. HW and YiZ wrote and revised the main manuscript. All authors contributed to the article and approved the submitted version.

## Funding

This study was supported by grants from the Key R&D Projects of Zhejiang Province (2022C02015), the Natural Science Foundation of Zhejiang Province (Z19C170001), the National Key Research and Development Program of China (2017YFD0500502), and the Funds of Ten Thousand People Plan.

## Conflict of Interest

The authors declare that the research was conducted in the absence of any commercial or financial relationships that could be construed as a potential conflict of interest.

## Publisher’s Note

All claims expressed in this article are solely those of the authors and do not necessarily represent those of their affiliated organizations, or those of the publisher, the editors and the reviewers. Any product that may be evaluated in this article, or claim that may be made by its manufacturer, is not guaranteed or endorsed by the publisher.
