# Supplementary figures and images for: Lactic Acid Bacteria Mixture Isolated From Wild Pig Alleviated the Gut Inflammation of Mice Challenged by Escherichia coli
Source: Front Immunol. 2022 Jan 26;13:822754. doi: 10.3389/fimmu.2022.822754 (PMC8825813; doi:10.3389/fimmu.2022.822754)

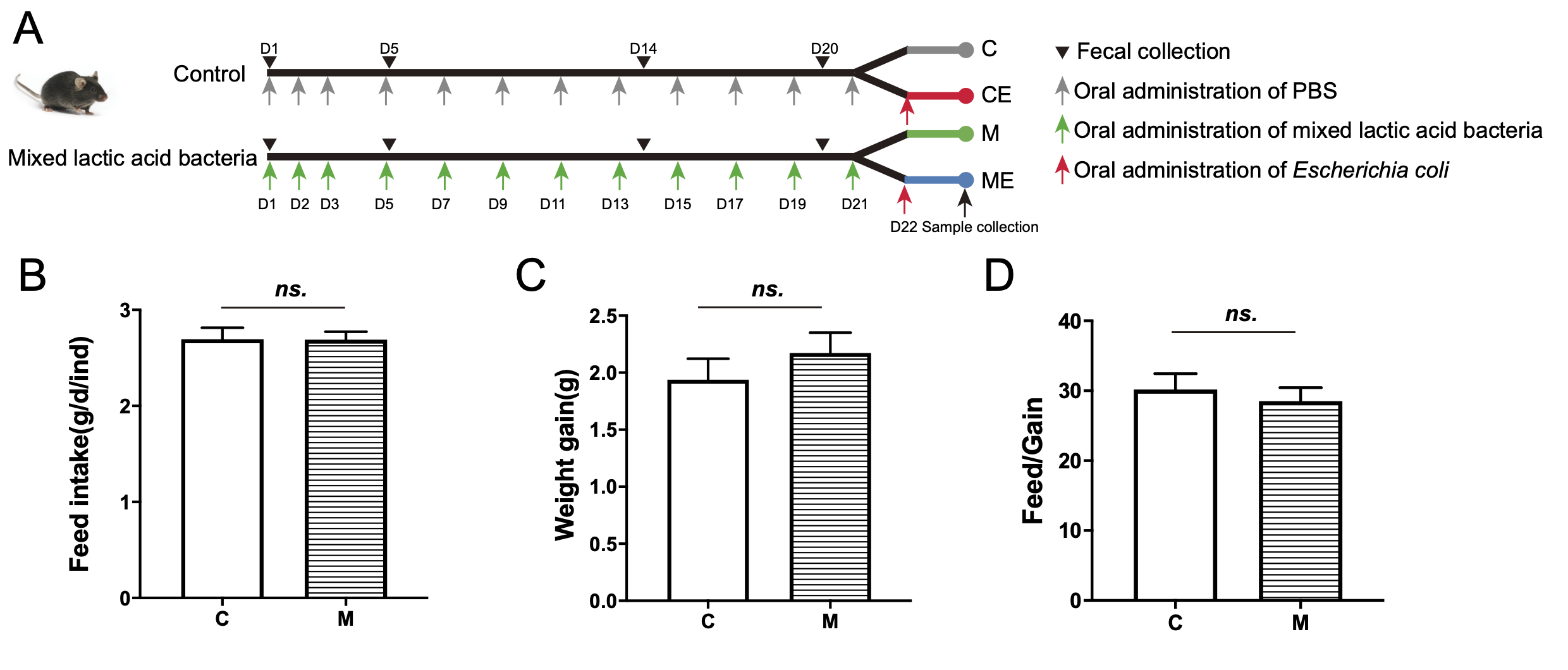

Supplement: Supplementary file 1 [file Image_1.tiff]
